# Crystal structures of 5,5′-bis(­hydroxy­methyl)-3,3′-biisoxazole and 4,4′,5,5′-tetrakis­(hydroxy­methyl)-3,3′-biisoxazole

**DOI:** 10.1107/S2056989018000828

**Published:** 2018-01-19

**Authors:** Rosario C. Sausa, Leah A. Wingard, Pablo E. Guzmán, Rose A. Pesce-Rodriguez, Jesse J. Sabatini, Peter Y. Zavalij

**Affiliations:** a US Army Research Laboratory, RDRL-WML-B, Aberdeen Proving Ground, MD 21005, USA; b US Army Research Laboratory, RDRL-WML-C, Aberdeen Proving Ground, MD 21005, USA; c University of Maryland, College Park, MD 20742, USA

**Keywords:** crystal structure, hy­droxy­methyl-biisoxazole, FTIR

## Abstract

The mol­ecular structure of 5,5′-bis(­hydroxy­methyl)-3,3′-biisoxazole, C_8_H_8_N_2_O_4_ (**1**), is composed of two *trans* planar isoxazole rings [r.m.s deviation = 0.006 (1) Å], each connected with a methyl hydroxyl group. Similarly, the structure of 4,4′,5,5′-tetrakis­(hydroxy­methyl)-3,3′-biisoxazole, C_10_H_12_N_2_O_6_ (**2**), is composed of two planar isoxazole rings [r.m.s. deviation = 0.002 (1) Å], but with four hydroxymethyl groups as substituents. Both mol­ecules sit on a center of inversion, thus *Z*′ = 0.5. The crystal structures are stabilized by networks of O—H⋯N [for (**1**)] and O—H⋯O hydrogen-bonding inter­actions [for (**2**)], giving rise to corrugated supra­molecular planes. The isoxazole rings are packed in a slip-stacked fashion, with centroid-to-centroid distances of 4.0652 (1) Å for (**1**) (along the *b*-axis direction) and of 4.5379 (Å) for (**2**) (along the *a*-axis direction).

## Abstract 2

Crystal structure, packing, and FTIR characterization of 5,5′-di­hydroxy­methyl-3,3′-biisoxazole and 4,4′,5,5′-tetra­hydroxy­methyl-3,3′-biisoxazole are reported.

## Chemical context   

The five-membered, heterocyclic isoxazole moiety forms the basis for a number of medical and agricultural products, as well as energetic materials (Galenko *et al.*, 2015 ▸; Sausa *et al.*, 2017 ▸; Wingard *et al.*, 2017*a* ▸,*b* ▸; Sysak & Obmińska-Mrukowicz, 2017 ▸). Its versatility stems from the electronegative oxygen and nitro­gen atoms, which provide the ring nucleophilic activity, and its three carbon atoms, which afford the addition of a variety of functional groups. The title compounds 5,5′-bis(hydroxy­methyl)-3,3′-biisoxazole (**1**) and 4,4′,5,5′-tetrakis(hydroxy­methyl)-3,3′-biisoxazole (**2**) exhibit two isoxazole rings, each attached with one or two hydroxymethy groups. These compounds have been synthesized recently in our laboratory as useful precursors to a new class of energetic materials. The addition of nitric acid to the title compounds results in nitrate esterification, yielding the energetic materials biisoxazole­bis(methyl­ene dinitrate) (**3**) and biisoxazole­tetra­kis­(methyl nitrate) (**4**), where a nitrate functional group replaces the hydrogen atom in the hydroxyl groups (Wingard *et al.*, 2017*a* ▸,*b* ▸). These derivative compounds are potential energetic plasticizing ingredients in nitro­cellulose or melt-castable formulations because the rings present Lewis-base behavior towards electrophilic nitro­cellulose and the alkyl nitric esters afford miscibility and compatibility with conventional energetic plasticizers.
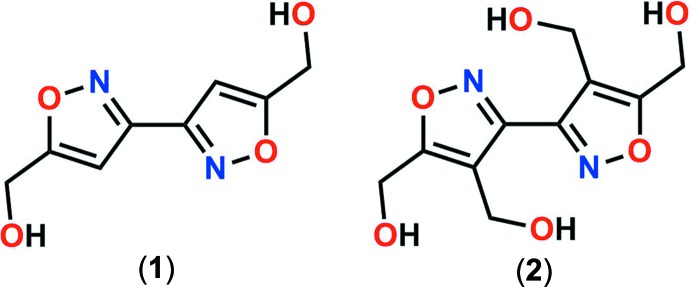


## Structural commentary   

**Figure 1:**
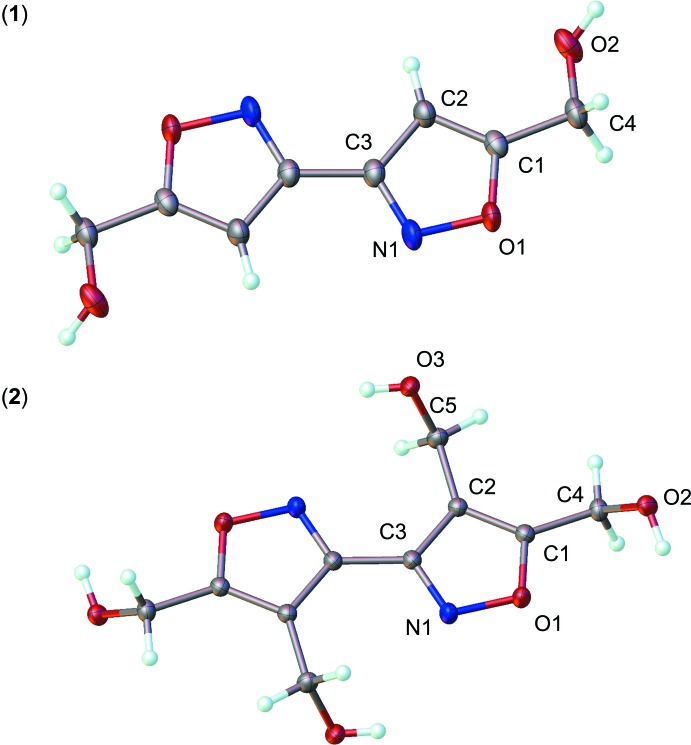
Mol­ecular conformation and atom-numbering scheme of compounds (**1**) and (**2**). Non-labeled atoms of both structures are generated by inversion (−*x* + 2, −*y* + 1, −*z* + 1). Non-hydrogen atoms are shown as 50% probability displacement ellipsoids.

**Figure 2:**
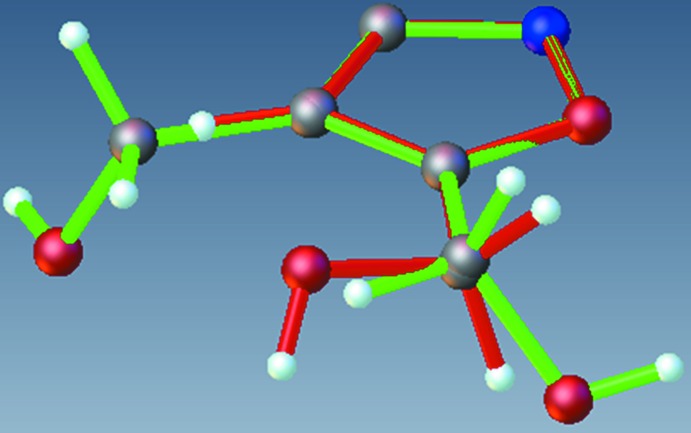
An overlay of the asymmetric units of compounds (**1**) and (**2**), depicted in red and green, respectively.

The title compounds exhibit mol­ecular structures typical of biisoxazole derivatives. Fig. 1 ▸ reveals that the isoxazole rings of (**1**) exhibit a *trans* planar configuration [r.m.s deviation = 0.0009 (1) Å], suggesting a delocalized aromatic π system. The C4 atom is nearly coplanar with the ring (atom-to-mean plane distance = 0.006 Å), whereas the C4—O2 bond is twisted slightly out of the plane, as evidenced by the torsion angles C2—C1—C4—O2 = −13.3 (2)° and O1—C1—C4—O2 = 167.55 (11)°. Atoms C1/C4/O2 form a plane that subtends a dihedral angle of 12.72 (1)° with respect to the isoxazole ring. Similarly, the isoxazole rings of (**2**) are nearly planar [r.m.s deviation = 0.002 (1) Å]; however, the corresponding O2—C4 bond is twisted more out of plane than that of compound (**1**), as evidenced by the magnitude of the torsion angle O2—C4—C1—O1 = −54.93 (11)°. For comparison, the torsion angle formed by atoms O3—C5—C2—C1 is −110.02 (11)°. The atoms O2/C4/C1 and O3/C5/C2 form planes subtending dihedral angles of 53.78 (8) and 69.37 (7)° with respect to the isoxazole ring. Superimposition of the ring atoms of both structures (see Fig. 2 ▸) yields an r.m.s. deviation of 0.01 Å. Finally, compound (**2**) exhibits a weak intra­molecular inter­action involving atoms O3—H3*A* and N1^iii^ [see Table 2 ▸ for the geometrical parameters; symmetry code: (iii) = −*x* + 2, −*y* + 1, −*z* + 1.]

## Supra­molecular features   

**Figure 3:**
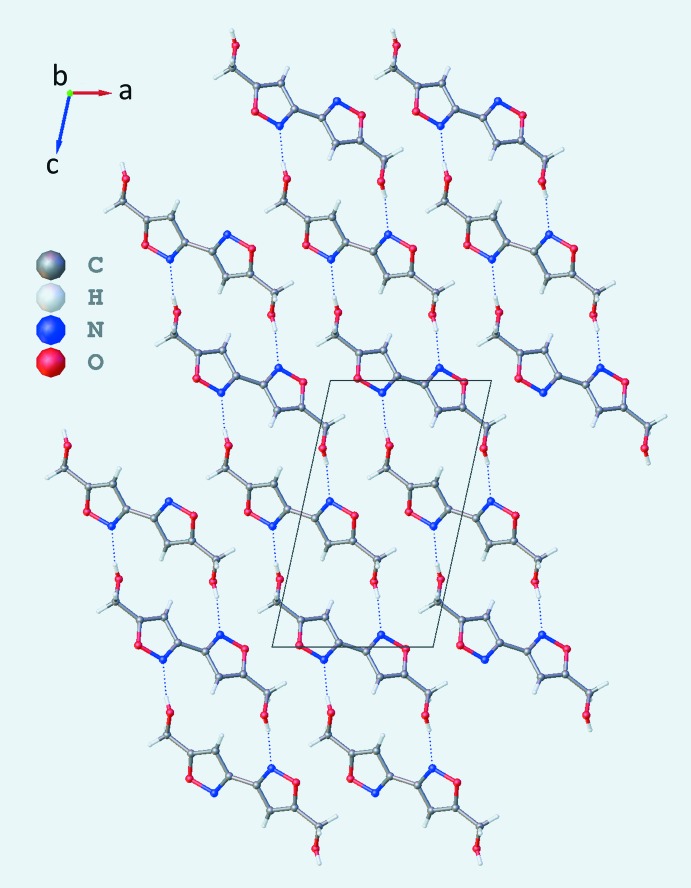
Crystal packing of (**1**) viewed along the *a-*axis direction. Dashed lines represent O2—H2*A*⋯N1^i^ hydrogen bonds; symmetry code: (i) *x* − 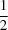, −*y* + 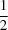, *z* − 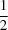.

**Figure 4:**
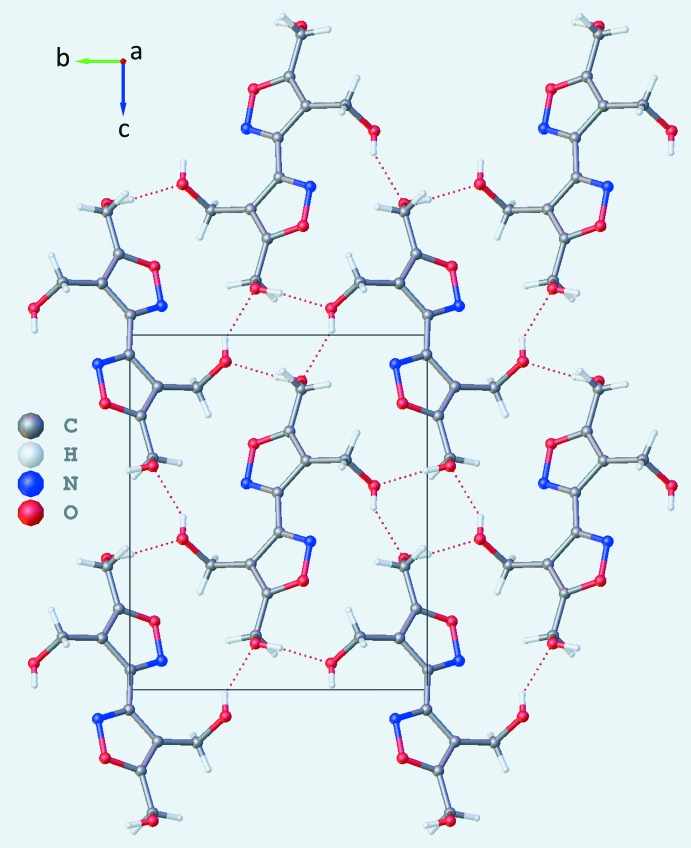
Crystal packing of (**2**) viewed along the *a-*axis direction. Dashed lines represent O2—H2*A*⋯O3^i^ and O3—H3*A*⋯O2^ii^ hydrogen bonds; symmetry codes: (i) −*x* + 1, *y* + 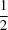, −*z* + 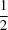; (ii) *x* + 1, −*y* + 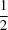, *z* + 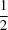.

Inter­molecular hydrogen bonding plays a key role in the stabilization of the crystal structures of the title compounds. Figs. 3 ▸ and 4 ▸ show the packing of (**1**) and (**2**), respectively, and Tables 1 ▸ and 2 ▸ list their hydrogen-bonding geometries. Compound (**1**) displays hydrogen bonding between the oxygen atoms O2, belonging to the hy­droxy groups, and the N1 atoms of the isoxazole rings of adjacent mol­ecules, generating a supra­molecular framework parallel to (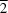
01) [O2⋯N1^i^ = 2.8461 (15) Å; symmetry code: (i) *x* − 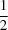, −*y* + 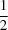, *z* − 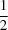]. In contrast, compound (**2**) forms a network of hydrogen bonds involving the hy­droxy groups O2—H2*A* and O3—H3*A* of adjacent mol­ecules, so that each OH group acts both as donor and acceptor [see Table 2 ▸ and Fig. 4 ▸; O2⋯O3^i^ = 2.694 (1) Å; symmetry code: (i) −*x* + 1, *y* + 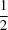, −*z* + 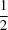; O3⋯O2^ii^ = 2.790 (1) Å; symmetry code: (ii) *x* + 1, −*y* + 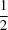, *z* + 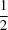]. In this way, each mol­ecule forms eight hydrogen bonds with the four closest surrounding analogues, giving rise to corrugated planes parallel to (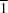
02).

The crystal structure of (**1**) reveals a slip-stacked geometry of the rings in the *b-*axis direction, with centroid-to-centroid distances of 4.0652 (1) Å and plane-to-plane shifts of 2.256 (2) Å. In contrast, in compound (**2**) the rings are stacked along the *a-*axis direction, with centroid-to-centroid distances of 4.5379 (4) Å and plane-to-plane shifts of 2.683 (2) Å.

## Database survey   

**Figure 5:**
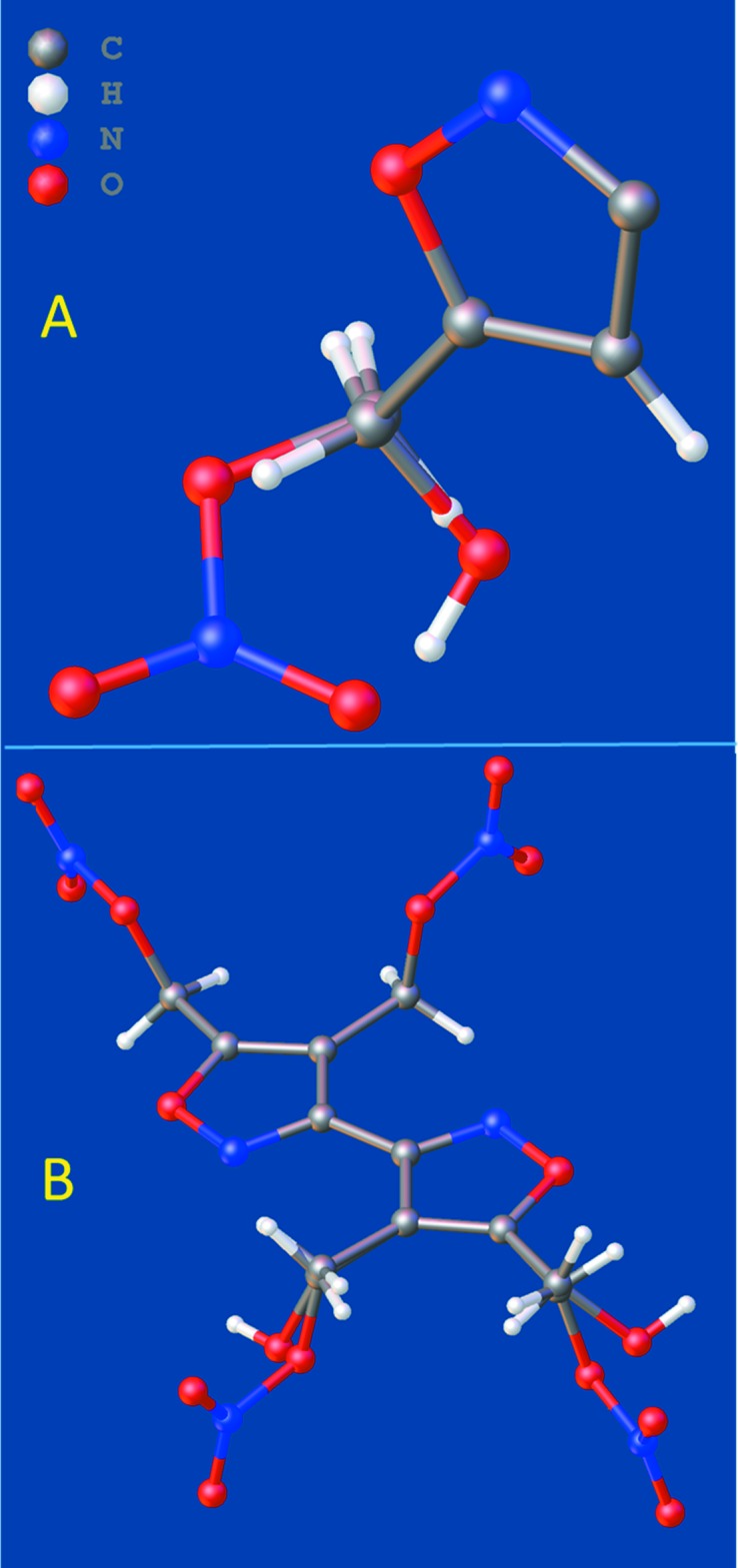
Overlays of the asymmetric units of (**1**) and (**3**) (A) and (**2**) and (**4**) (B).

A search of the Cambridge Structural Database (CSD web inter­face, December 2017; Groom *et al.*, 2016 ▸) and the Crystallography Open Database (Gražulis *et al.*, 2009 ▸) yielded the crystal structures of several compounds containing the biisoxazole moiety. For examples, see Cannas & Marongiu (1967 ▸) (CCDC 1111317, BIOXZL); van der Peet *et al.* (2013 ▸) (CCDC 935274, LIRLEF); Sausa *et al.* (2017 ▸) (CCDC 1540757, TAXDUU); Wingard *et al.* (2017*b* ▸) (CCDC 1529260, WANVEP). Compounds (**3**) (Sausa *et al.*, 2017 ▸) and (**4**) (Wingard *et al.* 2017*b* ▸) are noteworthy because they are nitrate derivatives of the title compounds (**1**) and (**2**), respectively, with the hydrogen atoms in the OH groups replaced by NO_2_ moieties. A superimposition of the respective isoxazole rings of compound (**1**) and (**3**) yields an r.m.s. deviation of 0.004 Å (Fig. 5 ▸A). In both mol­ecules, the rings adopt a *trans* conformations; however, in (**1**) the O1 and O2 atoms are in a *trans* conformation with respect to the C1—C4 bond, whereas in (**3**) the corresponding O atoms are in a *cis* conformation. In (**1**), the plane encompassing the atoms O2, C4, and C1 forms a dihedral angle of 12.72 (1)° with respect to the mean plane of the isoxazole ring, in contrast to a value of 66.8 (2)° in (**3**) for the corresponding atoms. A similar comparison between (**2**) and (**4**) yields an r.m.s. deviation of 0.01 Å for the superimposition of the isoxazole rings, and dihedral angles of 53.78 (8) and 69.37 (7)° for (**2**) (planes formed by the atoms O2/C4/C1 and O3/C5/C2, respectively) compared to those of 84.54 (14) and 84.81 (18)° or 79.19 (15) and 82.32 (17)° for (**4**) (Fig. 5 ▸B). The most striking supra­molecular difference between the title compounds and (**3**) and (**4**) is that the former exhibit hydrogen bonding, which contributes to the stability of their crystal structure.

## Synthesis and crystallization   

The synthesis of the title compounds has been reported recently (Wingard *et al.*, 2017**a* ▸,b* ▸). Briefly, they were prepared by [3 + 2] cyclo­addition of di­chloro­glyoxime and alcohol. In the case of compound (**1**), a saturated solution of sodium bicarbonate was added to a solution of di­chloro­glyoxime (30 g), propargyl alcohol (55.2 ml), and methanol (1900 ml) over 6 h. Once the reaction was complete, the product was stirred for an additional 10 h and the remaining solvent evaporated. A yield of 75% was obtained after the product was washed with distilled water, collected by Büchner filtration, and then dried. Compound (**2**) was prepared by adding dropwise a di­chloro­glyoxime and butyl alcohol solution (0.8 *M*) to a refluxing solution comprising NaHCO_3_ (6.7 g), 2-butyne-1,4-diol (13.72 g), and butyl alcohol (200 ml). Once the reaction was complete, the product was cooled to room temperature and the remaining solvent evaporated. Then, the product was washed with distilled water, filtered, and dried, resulting in a yield of 68%. Slow solvent evaporation of the title compounds in methanol yielded suitable single crystals for the X-ray diffraction experiments at 150K. We note the title compounds have nearly the same density (1.596 *vs* 1.597 Mg m^−3^), given that their mol­ecular mass and cell constants are quite different.

**Figure 6:**
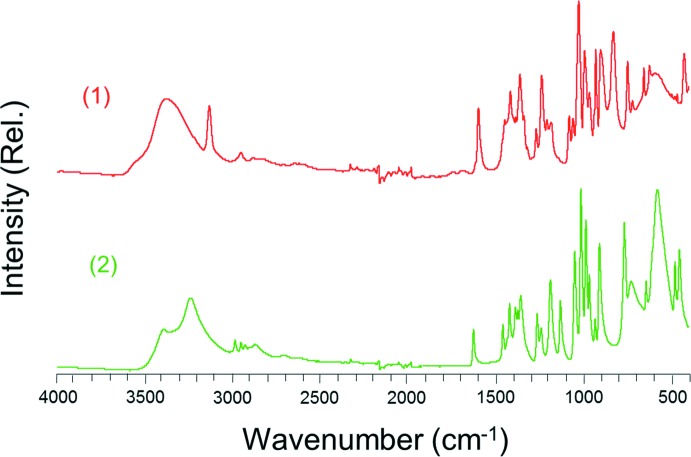
FTIR spectra of the title compounds.

Fig. 6 ▸ shows the FTIR spectra of (**1**) and (**2**) recorded with a Nicolet iS50 spectrophotometer, using attenuated total reflectance. The intense peak frequencies (cm^−1^) are listed as follows: Compound (**1**): 3371.83, 3126.65, 1596.96, 1415.14, 1360.62, 1268.13, 1237.16, 1080.70, 1058.61, 1026.40, 993.24, 929.53, 901.95, 828.87, 746.83, 653.69, 621.96, and 424.11. Compound (**2**): 3234.89, 1623.59, 1456.55, 1418.41, 1354.66, 1261.30, 1185.44, 1128.41, 1046.52, 1011.82, 984.07, 964.14, 931.24, 906.80, 764.50, 725.86, 641.00, 576.90, 475.85, and 449.97.

## Refinement   

Crystal data, data collection, structure solution and refinement details are summarized in Table 3 ▸. The hydrogen atoms for compound **(1**) were refined using a riding model with C—H = 0.93 or 0.98 Å and *U*_iso_(H) = 1.2*U*_eq_(C) and O—H = 0.74–0.85 Å and *U*_iso_(H) = 1.5*U*_eq_(O), whereas for compound (**2**) all the hydrogen atoms were refined independently including isotropic displacement parameters.

## Supplementary Material

Crystal structure: contains datablock(s) 1, 2. DOI: 10.1107/S2056989018000828/xi2007sup1.cif

Structure factors: contains datablock(s) 1, 2. DOI: 10.1107/S2056989018000828/xi20071sup3.hkl

Structure factors: contains datablock(s) 2. DOI: 10.1107/S2056989018000828/xi20072sup2.hkl

Supporting information file. DOI: 10.1107/S2056989018000828/xi20071sup4.cml

Supporting information file. DOI: 10.1107/S2056989018000828/xi20072sup5.cml

CCDC references: 1816712, 1816711

Additional supporting information:  crystallographic information; 3D view; checkCIF report

## Figures and Tables

**Table 1:** Hydrogen-bond geometry (Å, °) for (**I**)

*D*—H⋯*A*	*D*—H	H⋯*A*	*D*⋯*A*	*D*—H⋯*A*
O2—H2*A*⋯N1^i^	0.82	2.03	2.8461 (15)	171

Symmetry code: (i) 
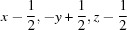
.

**Table 2:** Hydrogen-bond geometry (Å, °) for (**II**)

*D*—H⋯*A*	*D*—H	H⋯*A*	*D*⋯*A*	*D*—H⋯*A*
O2—H2*A*⋯O3^i^	0.849 (18)	1.849 (18)	2.6936 (11)	172.8 (16)
O3—H3*A*⋯O2^ii^	0.792 (19)	2.085 (19)	2.7898 (11)	148.3 (18)
O3—H3*A*⋯N1^iii^	0.792 (19)	2.550 (19)	3.0728 (12)	125.0 (16)

Symmetry codes: (i) 
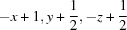
; (ii) 
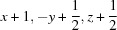
; (iii) 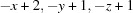.

**Table 3:** Experimental details

	(**I**)	(**II**)
Crystal data		
Chemical formula	C_8_H_8_N_2_O_4_	C_10_H_12_N_2_O_6_
*M*_r_	196.16	256.22
Crystal system, space group	Monoclinic, *P*2_1_/*n*	Monoclinic, *P*2_1_/*c*
Temperature (K)	150	150
*a*, *b*, *c* (Å)	7.7824 (3), 4.0652 (1), 13.2109 (5)	4.5379 (4), 9.9195 (8), 12.0177 (9)
β (°)	102.334 (4)	99.9312 (11)
*V* (Å^3^)	408.31 (2)	532.86 (8)
*Z*	2	2
Radiation type	Mo *K*α	Mo *K*α
μ (mm^−1^)	0.13	0.13
Crystal size (mm)	0.35 × 0.25 × 0.05	0.49 × 0.20 × 0.11

Data collection		
Diffractometer	Rigaku Oxford DiffractionSuperNova, Dualflex, EosS2	Bruker SMART APEXII CCD
Absorption correction	Multi-scan (*CrysAlis PRO*; Rigaku OD, 2015 ▸; Bourhis *et al.*, 2015 ▸)	Multi-scan (*SADABS*; Sheldrick, 2008 ▸)
*T*_min_, *T*_max_	0.207, 1.000	0.904, 0.985
No. of measured, independent and observed [*I* > 2σ(*I*)] reflections	3474, 823, 754	7638, 1737, 1570
*R*_int_	0.027	0.019
(sin θ/λ)_max_ (Å^−1^)	0.624	0.730

Refinement		
*R*[*F* ^2^ > 2σ(*F* ^2^)], *wR*(*F* ^2^), *S*	0.033, 0.086, 1.04	0.034, 0.072, 1.00
No. of reflections	823	1737
No. of parameters	66	106
H-atom treatment	H-atom parameters constrained	All H-atom parameters refined
Δρ_max_, Δρ_min_ (e Å^−3^)	0.28, −0.15	0.44, −0.22

Computer programs: *CrysAlis PRO* (Rigaku OD, 2015 ▸), *APEX2*, *XSHELL* and *SAINT* (Bruker, 2010 ▸), *SHELXT* (Sheldrick, 2015*a* ▸), *SHELXS97* (Sheldrick, 2008 ▸), *SHELXL2014* (Sheldrick, 2015*b* ▸), *OLEX2* (Dolomanov *et al.*, 2009 ▸), *Mercury* (Macrae *et al.*, 2008 ▸) and *PLATON* (Spek, 2009 ▸).

## supplementary crystallographic information

### 5,5'-Dihydroxymethyl-3,3'-biisoxazole (1) Crystal data

**Table:**

C_8_H_8_N_2_O_4_	*F*(000) = 204
*M**_r_* = 196.16	*D*_x_ = 1.596 Mg m^−^^3^
Monoclinic, *P*2_1_/*n*	Mo *K*α radiation, λ = 0.71073 Å
*a* = 7.7824 (3) Å	Cell parameters from 1980 reflections
*b* = 4.0652 (1) Å	θ = 2.8–26.2°
*c* = 13.2109 (5) Å	µ = 0.13 mm^−^^1^
β = 102.334 (4)°	*T* = 150 K
*V* = 408.31 (2) Å^3^	Block, colorless
*Z* = 2	0.35 × 0.25 × 0.05 mm

### 5,5'-Dihydroxymethyl-3,3'-biisoxazole (1) Data collection

**Table:**

Rigaku Oxford DiffractionSuperNova, Dualflex, EosS2 diffractometer	823 independent reflections
Radiation source: fine-focus sealed X-ray tube, Enhance (Mo) X-ray Source	754 reflections with *I* > 2σ(*I*)
Graphite monochromator	*R*_int_ = 0.027
Detector resolution: 8.0945 pixels mm^-1^	θ_max_ = 26.3°, θ_min_ = 2.8°
ω scans	*h* = −9→9
Absorption correction: multi-scan CrysAlisPro (Rigaku OD, 2015; Bourhis *et al.*, 2015)	*k* = −5→5
*T*_min_ = 0.207, *T*_max_ = 1.000	*l* = −16→16
3474 measured reflections	

### 5,5'-Dihydroxymethyl-3,3'-biisoxazole (1) Refinement

**Table:**

Refinement on *F*^2^	Hydrogen site location: inferred from neighbouring sites
Least-squares matrix: full	H-atom parameters constrained
*R*[*F*^2^ > 2σ(*F*^2^)] = 0.033	*w* = 1/[σ^2^(*F*_o_^2^) + (0.040*P*)^2^ + 0.183*P*] where *P* = (*F*_o_^2^ + 2*F*_c_^2^)/3
*wR*(*F*^2^) = 0.086	(Δ/σ)_max_ < 0.001
*S* = 1.03	Δρ_max_ = 0.28 e Å^−^^3^
823 reflections	Δρ_min_ = −0.15 e Å^−^^3^
66 parameters	Extinction correction: SHELXL-2016/4 (Sheldrick 2015), Fc^*^=kFc[1+0.001xFc^2^λ^3^/sin(2θ)]^-1/4^
0 restraints	Extinction coefficient: 0.041 (8)
Primary atom site location: dual	

### 5,5'-Dihydroxymethyl-3,3'-biisoxazole (1) Special details

**Table:**

Geometry. All esds (except the esd in the dihedral angle between two l.s. planes) are estimated using the full covariance matrix. The cell esds are taken into account individually in the estimation of esds in distances, angles and torsion angles; correlations between esds in cell parameters are only used when they are defined by crystal symmetry. An approximate (isotropic) treatment of cell esds is used for estimating esds involving l.s. planes.

### 5,5'-Dihydroxymethyl-3,3'-biisoxazole (1) Fractional atomic coordinates and isotropic or equivalent isotropic displacement parameters (Å^2^)

**Table:**

	*x*	*y*	*z*	*U*_iso_*/*U*_eq_	
C1	0.64217 (17)	0.3456 (3)	0.39844 (9)	0.0193 (3)	
C2	0.78918 (17)	0.5023 (3)	0.38810 (10)	0.0207 (3)	
H2	0.808128	0.621413	0.331370	0.025*	
C3	0.90869 (16)	0.4449 (3)	0.48325 (9)	0.0188 (3)	
C4	0.46651 (17)	0.3002 (3)	0.32830 (10)	0.0230 (3)	
H4A	0.453788	0.076229	0.302446	0.028*	
H4B	0.373826	0.343734	0.365286	0.028*	
N1	0.83872 (14)	0.2665 (3)	0.54646 (8)	0.0238 (3)	
O1	0.66636 (12)	0.2010 (2)	0.49285 (7)	0.0240 (3)	
O2	0.45577 (14)	0.5241 (3)	0.24526 (7)	0.0304 (3)	
H2A	0.412548	0.431590	0.190584	0.046*	

### 5,5'-Dihydroxymethyl-3,3'-biisoxazole (1) Atomic displacement parameters (Å^2^)

**Table:**

	*U*^11^	*U*^22^	*U*^33^	*U*^12^	*U*^13^	*U*^23^
C1	0.0199 (7)	0.0198 (7)	0.0165 (6)	0.0029 (5)	0.0004 (5)	−0.0032 (5)
C2	0.0194 (7)	0.0238 (7)	0.0174 (6)	0.0007 (5)	0.0009 (5)	−0.0005 (5)
C3	0.0173 (7)	0.0209 (7)	0.0174 (6)	0.0021 (5)	0.0019 (5)	−0.0029 (5)
C4	0.0190 (7)	0.0249 (7)	0.0226 (7)	−0.0005 (5)	−0.0009 (5)	−0.0054 (5)
N1	0.0173 (6)	0.0315 (7)	0.0198 (6)	−0.0029 (5)	−0.0019 (4)	−0.0002 (5)
O1	0.0180 (5)	0.0319 (6)	0.0199 (5)	−0.0052 (4)	−0.0008 (4)	0.0003 (4)
O2	0.0342 (6)	0.0275 (6)	0.0230 (5)	−0.0011 (4)	−0.0087 (4)	−0.0024 (4)

### 5,5'-Dihydroxymethyl-3,3'-biisoxazole (1) Geometric parameters (Å, º)

**Table:**

C1—C2	1.3419 (19)	C3—N1	1.3093 (17)
C1—C4	1.4901 (17)	C4—H4A	0.9700
C1—O1	1.3550 (15)	C4—H4B	0.9700
C2—H2	0.9300	C4—O2	1.4142 (17)
C2—C3	1.4143 (18)	N1—O1	1.4021 (14)
C3—C3^i^	1.465 (2)	O2—H2A	0.8200
			
C2—C1—C4	133.19 (12)	C1—C4—H4A	110.3
C2—C1—O1	110.24 (11)	C1—C4—H4B	110.3
O1—C1—C4	116.57 (11)	H4A—C4—H4B	108.5
C1—C2—H2	127.9	O2—C4—C1	107.28 (11)
C1—C2—C3	104.14 (11)	O2—C4—H4A	110.3
C3—C2—H2	127.9	O2—C4—H4B	110.3
C2—C3—C3^i^	129.00 (15)	C3—N1—O1	105.45 (10)
N1—C3—C2	111.97 (11)	C1—O1—N1	108.21 (10)
N1—C3—C3^i^	119.03 (14)	C4—O2—H2A	109.5
			
C1—C2—C3—C3^i^	179.69 (17)	C3—N1—O1—C1	0.23 (14)
C1—C2—C3—N1	−0.03 (15)	C4—C1—C2—C3	−179.05 (14)
C2—C1—C4—O2	−13.3 (2)	C4—C1—O1—N1	179.11 (11)
C2—C1—O1—N1	−0.26 (14)	O1—C1—C2—C3	0.18 (14)
C2—C3—N1—O1	−0.12 (15)	O1—C1—C4—O2	167.55 (11)
C3^i^—C3—N1—O1	−179.87 (14)		

Symmetry code: (i) −*x*+2, −*y*+1, −*z*+1.

### 5,5'-Dihydroxymethyl-3,3'-biisoxazole (1) Hydrogen-bond geometry (Å, º)

**Table:**

*D*—H···*A*	*D*—H	H···*A*	*D*···*A*	*D*—H···*A*
O2—H2*A*···N1^ii^	0.82	2.03	2.8461 (15)	171

Symmetry code: (ii) *x*−1/2, −*y*+1/2, *z*−1/2.

### 4,4',5,5'-Tetrahydroxymethyl-3,3'-biisoxazole (2) Crystal data

**Table:**

C_10_H_12_N_2_O_6_	*F*(000) = 268
*M**_r_* = 256.22	*D*_x_ = 1.597 Mg m^−^^3^
Monoclinic, *P*2_1_/*c*	Mo *K*α radiation, λ = 0.71073 Å
*a* = 4.5379 (4) Å	Cell parameters from 3988 reflections
*b* = 9.9195 (8) Å	θ = 2.7–32.1°
*c* = 12.0177 (9) Å	µ = 0.13 mm^−^^1^
β = 99.9312 (11)°	*T* = 150 K
*V* = 532.86 (8) Å^3^	Needle, colourless
*Z* = 2	0.49 × 0.20 × 0.11 mm

### 4,4',5,5'-Tetrahydroxymethyl-3,3'-biisoxazole (2) Data collection

**Table:**

Bruker SMART APEXII CCD diffractometer	1737 independent reflections
Radiation source: sealed tube	1570 reflections with *I* > 2σ(*I*)
Graphite monochromator	*R*_int_ = 0.019
Detector resolution: 8.333 pixels mm^-1^	θ_max_ = 31.3°, θ_min_ = 2.7°
φ and ω scans	*h* = −6→6
Absorption correction: multi-scan (SADABS; Sheldrick, 2008)	*k* = −14→14
*T*_min_ = 0.904, *T*_max_ = 0.985	*l* = −17→17
7638 measured reflections	

### 4,4',5,5'-Tetrahydroxymethyl-3,3'-biisoxazole (2) Refinement

**Table:**

Refinement on *F*^2^	Primary atom site location: structure-invariant direct methods
Least-squares matrix: full	Secondary atom site location: difference Fourier map
*R*[*F*^2^ > 2σ(*F*^2^)] = 0.034	Hydrogen site location: difference Fourier map
*wR*(*F*^2^) = 0.072	All H-atom parameters refined
*S* = 1.00	*w* = 1/[σ^2^(*F*_o_^2^) + (0.010*P*)^2^ + 0.3955*P*] where *P* = (*F*_o_^2^ + 2*F*_c_^2^)/3
1737 reflections	(Δ/σ)_max_ < 0.001
106 parameters	Δρ_max_ = 0.44 e Å^−^^3^
0 restraints	Δρ_min_ = −0.22 e Å^−^^3^

### 4,4',5,5'-Tetrahydroxymethyl-3,3'-biisoxazole (2) Special details

**Table:**

Geometry. All esds (except the esd in the dihedral angle between two l.s. planes) are estimated using the full covariance matrix. The cell esds are taken into account individually in the estimation of esds in distances, angles and torsion angles; correlations between esds in cell parameters are only used when they are defined by crystal symmetry. An approximate (isotropic) treatment of cell esds is used for estimating esds involving l.s. planes.

### 4,4',5,5'-Tetrahydroxymethyl-3,3'-biisoxazole (2) Fractional atomic coordinates and isotropic or equivalent isotropic displacement parameters (Å^2^)

**Table:**

	*x*	*y*	*z*	*U*_iso_*/*U*_eq_	
O1	0.58044 (17)	0.58291 (7)	0.30718 (6)	0.01981 (16)	
O2	0.23448 (16)	0.42300 (8)	0.13078 (6)	0.01890 (15)	
H2A	0.174 (4)	0.5035 (18)	0.1182 (14)	0.039 (4)*	
O3	0.93528 (19)	0.17777 (8)	0.42532 (7)	0.02252 (17)	
H3A	1.037 (4)	0.1801 (18)	0.4858 (16)	0.045 (5)*	
N1	0.7277 (2)	0.60707 (9)	0.41811 (7)	0.02010 (18)	
C1	0.6765 (2)	0.46292 (10)	0.27170 (8)	0.01576 (17)	
C2	0.8810 (2)	0.40630 (9)	0.35428 (8)	0.01513 (17)	
C3	0.9048 (2)	0.50244 (10)	0.44419 (8)	0.01585 (18)	
C4	0.5528 (2)	0.42337 (10)	0.15275 (8)	0.01753 (18)	
H4A	0.631 (3)	0.4856 (14)	0.1016 (12)	0.022 (3)*	
H4B	0.622 (3)	0.3337 (14)	0.1391 (11)	0.021 (3)*	
C5	1.0376 (2)	0.27405 (10)	0.35134 (9)	0.01875 (19)	
H5A	1.255 (3)	0.2866 (14)	0.3717 (12)	0.024 (3)*	
H5B	0.992 (3)	0.2362 (14)	0.2760 (11)	0.021 (3)*	

### 4,4',5,5'-Tetrahydroxymethyl-3,3'-biisoxazole (2) Atomic displacement parameters (Å^2^)

**Table:**

	*U*^11^	*U*^22^	*U*^33^	*U*^12^	*U*^13^	*U*^23^
O1	0.0240 (4)	0.0166 (3)	0.0163 (3)	0.0036 (3)	−0.0036 (3)	−0.0010 (3)
O2	0.0163 (3)	0.0169 (3)	0.0216 (3)	−0.0003 (3)	−0.0019 (3)	−0.0014 (3)
O3	0.0302 (4)	0.0154 (3)	0.0187 (4)	−0.0019 (3)	−0.0050 (3)	0.0026 (3)
N1	0.0253 (4)	0.0171 (4)	0.0153 (4)	0.0022 (3)	−0.0038 (3)	−0.0019 (3)
C1	0.0171 (4)	0.0143 (4)	0.0154 (4)	−0.0012 (3)	0.0015 (3)	−0.0003 (3)
C2	0.0167 (4)	0.0136 (4)	0.0148 (4)	−0.0008 (3)	0.0017 (3)	0.0001 (3)
C3	0.0183 (4)	0.0139 (4)	0.0145 (4)	−0.0012 (3)	0.0003 (3)	0.0001 (3)
C4	0.0171 (4)	0.0199 (4)	0.0147 (4)	−0.0007 (3)	0.0001 (3)	−0.0006 (3)
C5	0.0219 (4)	0.0161 (4)	0.0176 (4)	0.0030 (3)	0.0015 (3)	−0.0006 (3)

### 4,4',5,5'-Tetrahydroxymethyl-3,3'-biisoxazole (2) Geometric parameters (Å, º)

**Table:**

O1—N1	1.4050 (11)	C3—C2	1.4312 (13)
O2—H2A	0.849 (18)	C3—C3^i^	1.4657 (18)
O2—C4	1.4229 (12)	C4—C1	1.4952 (13)
O3—H3A	0.792 (19)	C4—H4A	0.980 (14)
N1—C3	1.3171 (12)	C4—H4B	0.966 (14)
C1—O1	1.3612 (12)	C5—O3	1.4354 (13)
C1—C2	1.3577 (13)	C5—H5A	0.982 (14)
C2—C5	1.4953 (13)	C5—H5B	0.969 (14)
			
C1—O1—N1	108.71 (7)	O2—C4—C1	112.33 (8)
C4—O2—H2A	108.4 (11)	O2—C4—H4A	110.6 (8)
C5—O3—H3A	110.4 (13)	O2—C4—H4B	108.3 (8)
C3—N1—O1	105.20 (8)	C1—C4—H4A	108.5 (8)
O1—C1—C4	116.17 (8)	C1—C4—H4B	108.9 (8)
C2—C1—O1	110.38 (8)	H4A—C4—H4B	108.1 (11)
C2—C1—C4	133.38 (9)	O3—C5—C2	111.30 (8)
C1—C2—C3	103.26 (8)	O3—C5—H5A	110.3 (8)
C1—C2—C5	127.83 (9)	O3—C5—H5B	106.3 (8)
C3—C2—C5	128.91 (9)	C2—C5—H5A	110.1 (8)
N1—C3—C2	112.45 (8)	C2—C5—H5B	109.7 (8)
N1—C3—C3^i^	118.88 (11)	H5A—C5—H5B	109.0 (11)
C2—C3—C3^i^	128.67 (11)		
			
O1—N1—C3—C2	−0.53 (11)	C1—C2—C5—O3	−110.02 (11)
O1—N1—C3—C3^i^	179.26 (10)	C2—C1—O1—N1	0.05 (11)
O1—C1—C2—C3	−0.35 (10)	C3—C2—C5—O3	68.82 (13)
O1—C1—C2—C5	178.72 (9)	C3^i^—C3—C2—C1	−179.21 (12)
O2—C4—C1—O1	−54.93 (11)	C3^i^—C3—C2—C5	1.74 (19)
O2—C4—C1—C2	128.39 (11)	C4—C1—O1—N1	−177.38 (8)
N1—C3—C2—C1	0.56 (11)	C4—C1—C2—C3	176.48 (10)
N1—C3—C2—C5	−178.49 (10)	C4—C1—C2—C5	−4.46 (18)
C1—O1—N1—C3	0.30 (10)		

Symmetry code: (i) −*x*+2, −*y*+1, −*z*+1.

### 4,4',5,5'-Tetrahydroxymethyl-3,3'-biisoxazole (2) Hydrogen-bond geometry (Å, º)

**Table:**

*D*—H···*A*	*D*—H	H···*A*	*D*···*A*	*D*—H···*A*
O2—H2*A*···O3^ii^	0.849 (18)	1.849 (18)	2.6936 (11)	172.8 (16)
O3—H3*A*···O2^iii^	0.792 (19)	2.085 (19)	2.7898 (11)	148.3 (18)
O3—H3*A*···N1^i^	0.792 (19)	2.550 (19)	3.0728 (12)	125.0 (16)

Symmetry codes: (i) −*x*+2, −*y*+1, −*z*+1; (ii) −*x*+1, *y*+1/2, −*z*+1/2; (iii) *x*+1, −*y*+1/2, *z*+1/2.
